# The Orphan Nuclear Receptor 4A1: A Potential New Therapeutic Target for Metabolic Diseases

**DOI:** 10.1155/2018/9363461

**Published:** 2018-06-14

**Authors:** Lei Zhang, Qun Wang, Wen Liu, Fangyan Liu, Ailing Ji, Yanzhang Li

**Affiliations:** Henan University School of Basic Medical Sciences, Henan University Joint National Laboratory for Antibody Drug Engineering, Kaifeng 475004, China

## Abstract

Orphan nuclear receptor 4A1 (NR4A1) is a transcriptional factor of the nuclear orphan receptor (NR4A) superfamily that has sparked interest across different research fields in recent years. Several studies have demonstrated that ligand-independent NR4A1 is an immediate-early response gene and the protein product is rapidly induced by a variety of stimuli. Hyperfunction or dysfunction of NR4A1 is implicated in various metabolic processes, including carbohydrate metabolism, lipid metabolism, and energy balance, in major metabolic tissues, such as liver, skeletal muscle, pancreatic tissues, and adipose tissues. No endogenous ligands for NR4A1 have been identified, but numerous compounds that bind and activate or inactivate nuclear NR4A1 or induce cytoplasmic localization of NR4A1 have been identified. This review summarizes recent advances in our understanding of the molecular biology and physiological functions of NR4A1. And we focus on the physiological functions of NR4A1 receptor to the development of the metabolic diseases, with a special focus on the impact on carbohydrate and lipid metabolism in skeletal muscle, liver, adipose tissue, and islet.

## 1. Introduction

The nuclear receptor superfamily includes at least 48 members of transcription factors that regulate multiple cellular and metabolic functions in diverse bioprocesses [1]. The NR4A family is an orphan nuclear receptor because the endogenous ligands of these receptors are not identified [2]. The NR4A protein subclass contains three highly homologous members named Nur77/NR4A1 (human homologue TR3, mouse homologue Nur77, and rat homologue NGFI-B), Nurr1/NR4A2, and Nor1/NR4A3 [3, 4]. These three NR4A receptors exhibit a high degree of homology in genomic structure [5], an *N*-terminal region that contains a ligand-independent activation function-1 (AF-1), also known as the transactivation domain (TAD), which is required for the coactivator recruitment and transcriptional activity [6], a conserved central DNA-binding domain (DBD) composed of 2 zinc fingers that recognize specific DNA sequences, a hinge region, and a ligand-binding domain (LBD) that contains a ligand-dependent AF-2 transactivation domain in the C-terminal region [7, 8]. The AF-1 domain is necessary for the recruitment of other transcription factors and transcriptional activity in the *N*-terminal region [6]. The transcriptional activation activity of NR4A receptors is exerted as monomers at specific DNA sequences known as NGFI-B response element (NBRE), which consist of an octanucleotide (AAAGGTCA) motif [9–11]. NR4A receptors also bind as homodimers or heterodimers to a Nur-responsive element (NuRE:TGATATTACCTCCAAATGCCA) which has been characterized from the proopiomelanocortin gene promoter [11]. In addition, both NR4A1 and NR4A2 are able to bind as heterodimers with the 9-cis RAR to an imperfect direct repeat (DR) sequence separated by five nucleotides (DR-5: GGTTCACCGAAAGGTCA) [12, 13]. The LBD structure of the NR4A1 seems clear, but its ligand binding and regulation activity are controversial. The LBDs recognize small molecule ligands to induce the characteristic responses. Heptad repeats of hydrophobic amino acids in LBD are involved in nuclear receptor dimerization and may regulate target nuclear localization [6]. However, structural studies on the LBD of the three NR4A members reveal that the hydrophilic surfaces, instead of the classic hydrophobic cleft that recruits the coactivator, are contained in the LBD of the NR4A receptor [14]. Therefore, no appropriate ligand-binding cavity is used to bind small molecular ligands and regulate the physiological function of NR4A. These observations suggest that the regulation activity of NR4A receptors is ligand independent and the function of NR4A is dependent on receptor expression and posttranslational modification [15]. However, several ligands that may interact with these receptors are identified recently [16–18]. Therefore, it is not clear whether NR4A is still a ligand-independent orphan nuclear receptor. More experimental data are required to provide a more appropriate answer.

NR4A1 is the most deeply research member of the NR4A family, and there is an overlapping and compensatory biological function between NR4A1 and the other two members from the NR4A family because of the structural homology. NR4A1 is an early response gene with pleotropic functions that are rapidly and transiently induced by a diverse range of signals, including stressors [19], growth factors, cytokines [20], the proanorexic melanocortin *α*-MSH [21, 22], glucose, fatty acids [4], lipopolysaccharides [23], exercise [24], and many small molecular compounds. Many physiological and pathological processes are related to NR4A1 expression, such as glucose and lipid metabolism, insulin synthesis, vascular disease, inflammation, immunity, and carcinogenesis control [25–27]. NR4A1 is associated with various hormonal, physiological, and pathophysiological diseases in humans, including metabolic disease [28, 29], cardiovascular disease [30], apoptosis [31], neurodegenerative diseases [32], steroidogenesis [30, 33], inflammation [34, 35], and oncogenesis [36]. This review summarizes recent progress in our understanding of the physiological and pathophysiological functions of NR4A1 receptors and discusses the role of these receptors as transcriptional regulators of gene expression in glucose and lipid metabolism.

## 2. Diverse Stimuli and Signals Induce NR4A1 Expression

NR4A1 is an immediate-early response gene that is induced by a diverse variety of signals in a wide range of tissues and cultured cells. Physiological stressors also induce NR4A1 expression and activation. Fasting induces hepatic NR4A1, which may bind to the putative NBRE of the fibroblast growth factor-21 (FGF21) promoter and regulate FGF21 expression, which plays pivotal roles in the treatment of metabolic syndromes [37]. A 17-fold increase of NR4A1 is observed in cardiomyocytes during acute ischemia/reperfusion (I/R) in dog, and the overexpression of NR4A1 significantly increases proteins translocated to the mitochondria. Therefore, NR4A1 may be involved in I/R-induced cell death and mitochondrial damage [38–40]. The following stimuli also induce NR4A1 in different cells: drugs of abuse, stress in nuclei of the motivation/reward circuit of the brain [41], membrane depolarization in PC12 pheochromocytoma cell line [42], mechanical agitation in several leukemic cell lines in a stimulus-dependent manner [43], acute hypotensive hemorrhage, intravenous injection of interleukin-1 beta, chronic salt loading, and acute bilateral adrenalectomy in similar neuroendocrine cell types [44].

NR4A1 is an orphan nuclear receptor, and no traditional ligand-binding cavity is used to bind small molecular ligands and regulate the physiological functions of NR4A1. No endogenous ligands have been previously identified for NR4A1. However, several synthetic and natural compounds that may interact with NR4A1 in unconventional ways are identified as agonists or antagonists. Cytosporone B (Csn-B) is the first naturally occurring agonist for nuclear orphan receptor NR4A1. Csn-B binds with high affinity (IC50 = 0.278 nM) to the ligand-binding domain of NR4A1 and stimulates NR4A1-dependent activities [45, 46]. Indeed, Csn-B physically binds to NR4A1 resulting in an increased expression and stimulation of its transcriptional activity *in vivo* [47]. The first NR4A1 agonist, 6-mercaptopurine, is obtained through high-throughput screening, and it inhibits tumor necrosis factor-*α* production in microglia via NR4A1-mediated transrepression and PI3K/Akt/mTOR signaling-mediated posttranslational modification. This agonist may be a good candidate for the effective treatment of nerve inflammation-related neurodegenerative diseases [27, 35]. Celastrol is another NR4A1 agonist that alleviates inflammation and induces autophagy in a NR4A1 dependent via interaction with TRAF2 in primary embryonic fibroblasts and C57 mice [48]. The following small molecular compounds also directly regulate NR4A1 activity in different cells: 1,1-bis(3′-indolyl)-1-(p-hydroxyphenyl)methane (DIM-C-pPhOH) and the related p-carboxymethylphenyl (1,1-bis(3′-indolyl)-1-(p-carboxymethylphenyl)methane (DIM-C-pPhCO2 Me)) analogs, which bind NR4A1 to inhibit growth and induce apoptosis in several cancer cell lines and tumors from mouse xenografts [49, 50]. Amoitone B is also a natural agonist to NR4A1, and it exhibits strong antitumor activity *in vivo* and *in vitro* [51].

Small molecular compounds also modulate NR4A1 activity by influencing the upstream signaling pathway of NR4A1. Retinoic acids induce NR4A1 expression and apoptosis, which is completely dependent on NR4A1 in mouse thymocytes [52]. The pronounced increase in NR4A1 expression by palmitate and oleate plays a pivotal role in the adaptive process of hyperlipidemia in beta-cells (INS cells) [53]. The following small molecule compounds also regulate NR4A1 activity: the *β*-adrenergic agonist isoproterenol in cardiomyocytes [54–56], trichostatin A (TSA) in PC12 cells [57], glucagon and forskolin in vivo and *in vitro* [58], norcantharidin (NCTD) in melanoma cells [59], phorbol ester (TPA) in granulosa cells [60], and thiazolidinedione drugs in 3T3-L1 adipocytes [61].

The orphan nuclear receptor NR4A1 regulates diverse cellular activities, and the nature of these biological functions is dependent on NR4A1 expression levels and the physiological context. Numerous growth promoter signals, such as insulin-like growth factor binding protein-3 (IGFBP-3), nerve growth factor (NGF), epidermal growth factor (EGF), and vascular endothelial growth factor (VEGF), induce NR4A1 expression. The elevated expression of NR4A1 induced by the *β*-cell transcription factor Nkx6.1 promotes islet *β*-cell proliferation and plays an important role in the functional restoration of these cells [62–64]. NR4A1 mRNA and protein expression induced by platelet-derived growth factor (PDGF) via ERK-MAPK-dependent signaling pathways is involved in the regulation of vascular smooth muscle cell proliferation [65]. The following growth promoter signals are also relevant for NR4A1 activity: IGFBP-3 in chondrocytes [66], vascular endothelial growth factor-A (VEGF-A) in endothelial cells [67], NGF in PC12 cells, and EGF in PC12 cells [68].

NR4A1 plays a critical role in numerous cellular processes in response to inflammatory-related cytokines, such as lipopolysaccharide (LPS), TNF*α*, IL-1, and oxidized lipids [69, 70]. Recent interest has been focused on the potent anti-inflammatory effect of NR4A1 in inflammatory diseases [71, 72]. Genetic studies revealed a critical role of NR4A1 in the control of inflammatory responses, which is highlighted by its protective function in atherosclerosis and obesity [48].

The role of microRNAs (miRNAs) is a far-reaching discovery in cancer in the past decade. miRNAs act as negative regulators of gene expression via combining to the 3′ untranslated region (3′ UTR) of a target mRNA to destabilize it or inhibit its translation [36]. miR-124 targets NR4A1 and decreases its expression and function, which attenuates cell proliferation in DAOY medulloblastoma cells [36].

## 3. NR4A1 Receptor in Carbohydrate Metabolism

**Figure 1:**
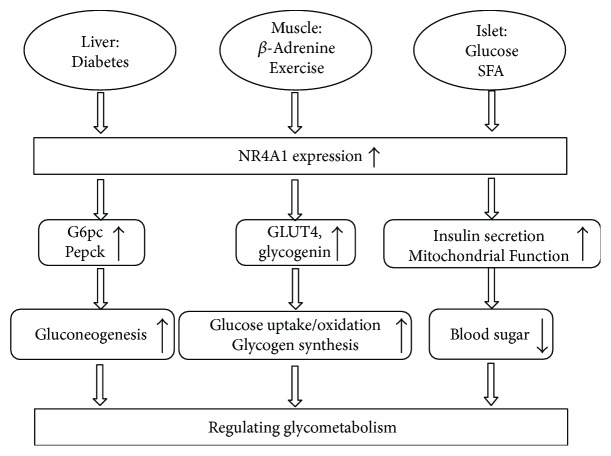
Summary of the studies describing tissue-specific NR4A1 activity in carbohydrate metabolism.

Glycogen synthesis, decomposition, and gluconeogenesis in the liver play pivotal roles in the regulation of glucose homeostasis. The dysregulation of these processes may contribute to the pathogenesis of several diseases, including diabetes mellitus, obesity, and hepatic steatosis. The expression of NR4A1 is closely related to gluconeogenesis. NR4A1 is highly expressed in type 1 diabetes mellitus (T1DM) and type 2 diabetes mellitus (T2DM) mouse models, and the ablation of liver NR4A1 in T2DM db/db mice via adenoviral delivery of a dominant-negative NR4A1 inhibits the expression of key enzymes of gluconeogenesis in the liver and restores elevated glucose levels to near normal [73, 74]. NR4A1 binds and sequesters liver kinase B1 (LKB1) in the nucleus and prevents LKB1 translocation from the nucleus to the cytosol to phosphorylate adenosine 5′-monophosphate- (AMP-) activated protein kinase *α* (AMPKα), which regulates the expression of key enzymes of liver gluconeogenesis, such as glucose-6-phosphatase catalytic subunit (G6pc) and phosphoenolpyruvate carboxykinase (Pepck), and blood sugar levels [18]. These data demonstrate that NR4A1-mediated gluconeogenesis plays an important role in the manifestation of diabetic hyperglycemia. Moreover, NR4A1 suppresses hepatocellular carcinoma via switching glucose metabolism toward gluconeogenesis through attenuating Pepck sumoylation [75]. Conversely, reduced NR4A1 receptor expression markedly decreases hepatic glucose production and lowers blood glucose levels [74]. Hepatic glucose production and liver insulin resistance are reduced significantly, and systemic glucose metabolism is altered in mice lacking NR4A1 fed a high-fat diet [27, 76]. The NR4A1 agonist Csn-B increases hepatic glucose production and blood glucose levels in fasting mice [16]. In addition, berberine, an oral antidiabetic drug, has been shown to activate adenosine 5′-monophosphate- (AMP-) activated protein kinase (AMPK) and increase hepatic FGF21 expression via NR4A1. Therefore, FGF21 may be a target gene of NR4A1 and exhibit multiple beneficial effects on energy metabolism [77, 78].  Figure 1 provides a schematic overview of the impact of NR4A1 on carbohydrate metabolism in the liver.

Carbohydrate is primarily stored in the form of glycogen in skeletal muscle. Most glycogen is deposited and stored in human skeletal muscle, which accounts for 70–80% of total glycogen. The liver stores the remaining portion of glycogen, which accounts for 20% of total glycogen. Another negligible but physiologically significant portion of glycogen is stored in the cardiac muscle and brain [79]. Skeletal muscle plays an irreplaceable role in the preservation of blood sugar and the regulation of blood glucose levels. The NR4A1 receptor is closely related to skeletal muscle glucose metabolic processes, including glucose uptake, glucose oxidation, glycogen synthesis, and skeletal muscle growth. And numerous stimuli, such as insulin, exercise, and beta-adrenergic signals, induce NR4A1 expression in skeletal muscle [80–82]. Beta-adrenergic signaling regulates glucose metabolism in skeletal muscle, and beta-adrenergic agonists, such as isoprenaline [83] and Csn-B, exquisitely mediate NR4A1 expression [16]. Therefore, the NR4A1 receptor is a potential mediator of skeletal muscle glucose metabolism via beta-adrenergic signaling. A NR4A1-coding adenovirus is used to infect L6 rat skeletal muscle myotubes and overexpress NR4A1 in skeletal muscle of chow-fed and fat-fed rats to examine the contribution of the NR4A1 receptor to skeletal muscle basal glucose uptake. This overexpression significantly increases basal glucose uptake and promotes the expression of genes associated with glucose uptake, such as glucose transporter 4 (GLUT4) and glycogenin [80]. These data provide compelling evidence that NR4A1 is a functional regulator of glucose uptake in skeletal muscle. NR4A1 in skeletal muscle also regulates genes involved in glycogen synthesis. Transgenic overexpression of NR4A1 remarkably increases glucose oxidation and glycogen synthesis and enhances glycogen synthase, hexokinase, and phosphofructokinase activity in L6 skeletal muscle cells and the skeletal muscle of chow- and fat-fed rats [79, 80]. NR4A1 is also closely related to glucose utilization under specific stimulation, including insulin [76, 84], endurance exercise [24, 85], and local electrical stimulation [24]. High-fat diet (60% calories from fat) fed mice with genetic deletion of NR4A1 exhibit increased susceptibility to diet-induced obesity and insulin resistance [76]. Conversely, insulin could upregulate NR4A1 expression significantly in cultured L6 skeletal muscle cells. However, the upregulated NR4A1 is localized in the nucleus and is not translocated to the cytoplasm [86]. Endurance exercise also upregulates NR4A1 receptor expression. A recent study demonstrates that regular physical exercise upregulates NR4A1 and related glucose metabolic gene expression in skeletal muscle [85]. Rats are performed acute 3 h low-intensity swimming or 3 h low-intensity treadmill running to investigate the role of local contractile activity on NR4A1 receptor expression in skeletal muscle during exercise. The results demonstrate that low-intensity swimming and treadmill running increase NR4A1 expression in muscle. Conversely, acute 1 h local electrical stimulation to the motor nerves of resting rats upregulates NR4A1 expression in skeletal muscle. These results suggest that local muscle contractile activity is required to increase NR4A1 expressions during exercise [24]. The expression of NR4A1 in skeletal muscle significantly enhances mitochondrial function in type 2 diabetes [33]. NR4A1 expression also promotes myocyte proliferation and skeletal muscle growth. Genetic knockout of NR4A1 impairs muscle growth during developmental myogenesis and muscle regeneration in mice [33, 81]. These results demonstrate that NR4A1 is a potential mediator of neuromuscular signaling in controlling glucose metabolic gene expression.  Figure 1 provides a schematic overview of the impact of NR4A1 on carbohydrate metabolism in skeletal muscle.

Insulin and glucagon secreted from the islets of Langerhans regulate blood sugar balance, and the malfunction will lead to various diseases, such as type 2 diabetes, which is influenced by genetic backgrounds and environmental factors [4]. NR4A1 can be induced by glucose [87] and saturated fatty acids (SFA) in pancreatic *β*-cells and regulates cell proliferation and insulin secretion [4, 88]. Therefore, the NR4A1 receptor is also a lipotoxicity sensor. Pancreatic *β*-cells are exposed to high concentrations of palmitate [4] or glucose to investigate the effect of glucose and saturated fatty acids on insulin secretion [87]. The results demonstrate that palmitate and glucose upregulated NR4A1 expression and insulin secretion [53]. These results have implications for the link between NR4A1 expression and insulin secretion in pancreatic *β*-cells. Moreover, knockdown of NR4A1 and NR4A3 impedes the production of ATP and ultimately inhibits glucose-stimulated insulin secretion. In addition, NR4A1 and NR4A3 are critical for *β*-cell mitochondrial function and insulin secretion [89]. NR4A1 induces Nkx6.1 and Cdk5r1 expression, which mediate *β*-cell proliferation, and promotes *β*-cell mass to reverse this hallmark of T1DM and T2DM [62–64]. NR4A1 promoter is hypermethylated in patients with T2DM and in a mouse model of T2DM. T2DM model mice treated with the DNA methyltransferase 1 inhibitor aurintricarboxylic acid (ATA) show reduced activation of DNA methyltransferase 1 in pancreatic *β*-cells and increased expression of NR4A1 and decreased blood glucose [90]. In summary, NR4A1 expression plays an important role in the adaptive process of beta-cells to hyperglycemia.  Figure 1 provides a schematic overview of the impact of NR4A1 on carbohydrate metabolism in islets.

## 4. NR4A1 Receptor in Lipid Metabolism

**Figure 2:**
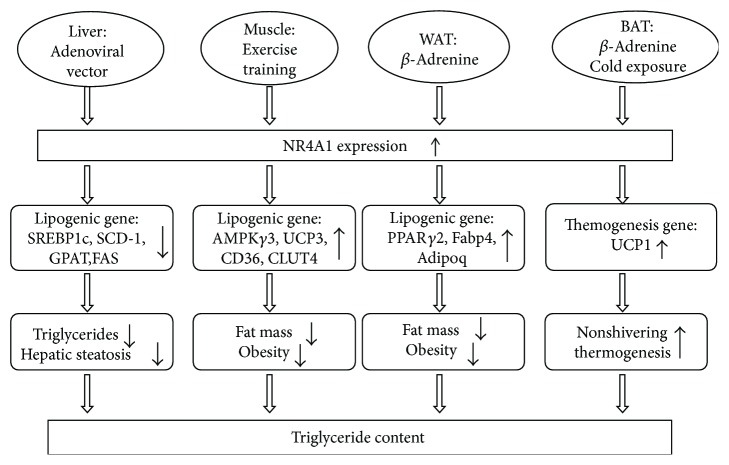
Summary of the studies describing tissue-specific NR4A1 activity in lipid metabolism.

The NR4A1 receptor is also involved in lipid metabolism in the liver. The abnormal expression of NR4A1 in the liver is implicated in numerous pathophysiological processes, including fat metabolism, cholesterol metabolism, and hepatic steatosis. Hepatic NR4A1 overexpressing mice are generated using adenoviral vectors, and modulation of the plasma lipid profile is established, such as cluster of differentiation 36 (CD36) and stearoyl-coA desaturase-1 (SCD-1), to examine the contribution of the NR4A1 receptor to hepatic lipid metabolism [91, 92]. The results demonstrate that NR4A1 overexpression attenuates the production of hepatic triglycerides and regulates a variety of key genes involved in lipid metabolism. NR4A1 inhibits sterol regulatory element-binding protein 1c (SREBP1c) expression, which further reduces target gene expression, including SCD-1, mitochondrial glycerol-3-phosphate acyltransferase (GPAT), fatty acid synthase (FAS), and the LDL receptor [92]. NR4A1 modulates hepatic lipid metabolism via suppression of SREBP1c activity. The liver is an important organ in the balance of cholesterol metabolism, and NR4A1 is implicated in hepatic cholesterol metabolism. Zhang et al. use siRNA to attenuate NR4A1 and transfection of the recombinant plasmid to increase NR4A1 in HepG2 cells to investigate the underlying mechanism. NR4A1 regulates hepatic cholesterol metabolism via suppression of low-density lipoprotein receptor (LDLR) and 3-hydroxy-3-methylglutaryl-coenzyme A reductase (HMGCR) expression [93]. However, high cholesterol levels upregulate NR4A1 expression [94]. Hepatic steatosis and the expression of lipogenic genes increase significantly in NR4A1 null mice fed a high-fat diet [76]. In summary, NR4A1 plays an important role in the regulation of liver fat content and lipid metabolism.  Figure 2 provides a schematic overview of the impact of NR4A1 on lipid metabolism in the liver.

The NR4A1 receptor is also involved in lipid metabolism in muscle. NR4A1 plays an important metabolic role in modulating lipolysis in muscle. Attenuation of NR4A1 expression by siRNAs in C2C12 skeletal muscle cells decreases lipolysis and the expression of key genes and proteins involved in the regulation of energy expenditure and lipid homeostasis, including adenosine 5′-monophosphate- (AMP-) activated protein kinase gamma 3 (AMPK*γ*3), uncoupling protein 3 (UCP3), CD36, adiponectin receptor 2, GLUT4, and caveolin-3 [56]. Female NR4A1-deficient mice fed a high-fat diet exhibit higher weights and fat mass compared to wild-type mice fed a high-fat diet [95]. Mitochondrial dysfunction is an important hallmark of the pathogenesis of T2DM, and oxidative metabolism and mitochondrial activity are increased in NR4A1-overexpressing transgenic muscle mice [33]. Notably, NR4A1 expression in the muscle of obese men is significantly lower than that of lean men, and the expression is closely correlated with body-fat content and insulin sensitivity [80]. Therefore, low NR4A1 expression impairs lipolysis and causes fat accumulation which results in obesity. Impaired glucose and lipid metabolism are observed in the skeletal muscle of low capacity rats, and exercise training reversed this situation, which improves glucose and lipid metabolism and increases the expression of NR4A1 and target genes, including *β*2 adrenergic receptor (*β*2-AR), GLUT4, UCP3, and FAT/CD36, in skeletal muscle [96, 97].  Figure 2 provides a schematic overview of the impact of NR4A1 on lipid metabolism in muscle.

NR4A1 also plays an important metabolic role in the modulation of lipid metabolism in adipose tissue. Adipose tissue is essential in the regulation of body weight, and NR4A1 is implicated in the regulation of fat content. Higher weight and fat mass are observed in NR4A1-deficient mice fed a high-fat diet compared to wild-type mice, which supports the function of NR4A1 in mediating lipolysis and energy expenditure. NR4A1 expression is significantly decreased in mouse models of obesity and diabetes [84]. *β*-Adrenergic stimulation and fasting induce NR4A1 expression in white adipose tissue, and *β*-adrenergic stimulation also induces the expression of peroxisome proliferator-activated receptor gamma 2 (PPAR*γ*2). The expression of several known PPAR*γ*2 targets is also found among NR4A1-regulated genes, such as G0/G1 switch 2 (G0s2), fatty acid-binding protein 4 (Fabp4), and adiponectin (Adipoq). These results suggest an overlapping biological function between NR4A1 and PPAR*γ*2 [20]. The PPAR*γ*2 promoter is a direct target of NR4A1-dependent repressive regulation, and the *N*-terminal domain of NR4A1 is required for this regulation [20]. Brown adipose tissue shares a common developmental ancestry with skeletal muscle, and it is a specialized fat tissue that is dedicated to nonshivering thermogenesis, which is regulated by adrenoceptor antagonists and cold exposure [98, 99]. Adrenoceptor antagonist and cold exposure have upregulated NR4A1 expression in brown adipose tissue. Therefore, NR4A1 is implicated in the nonshivering thermogenesis of brown adipose tissue [100]. A dominant-negative mutant NR4A1 receptor that prevented the transcriptional activity of all NR4A receptors is transfected to brown preadipocytes *in vitro* to investigate the contribution of NR4A1 to nonshivering thermogenesis in brown adipose tissue [101]. Attenuation of NR4A receptor activity inhibited beta-adrenoceptor receptor-stimulated uncoupling protein 1 (UCP1) gene transcription *in vitro* [88]. UCP1 was identified as a direct target of Nor-1 in gel shift, chromatin immunoprecipitation, and luciferase-reporter assay promoter analyses in brown adipose tissue [101]. NR4A1 is implicated in adipogenesis, and treatment of 3T3-L1 preadipocytes with an adipogenic cocktail rapidly upregulates NR4A1 [72]. However, NR4A1 induction is not an obligatory feature of preadipocyte adipogenesis because the adipogenesis of 3T3-L1 or 3T3-F442A preadipocytes is inhibited by the retroviral transduction of NR4A1 [72]. To investigate the contribution of NR4A1 to adipogenesis, NR4A1 is overexpressed and repressed in 3T3-L1 preadipocytes. Chronic overexpression and chronic repression of NR4A1 inhibit adipogenesis, whereas transient overexpression of NR4A1 significantly promoted adipogenesis [102]. These data suggest that only a transient induction of NR4A1 may be required for adipogenesis in 3T3-L1 preadipocytes.  Figure 2 provides a schematic overview of the impact of NR4A1 on lipid metabolism in adipose tissue.

In addition, the NR4A receptors have been associated to a wide range of pathological conditions, such as cancer, inflammation, neurological or cardiovascular diseases, and immune alterations. For cancer cells, NR4A1 acts as a survival factor in the nucleus, but it transforms into a killer when immigrated to mitochondria [103, 104]. In the mitochondria, NR4A1 can interact with B-cell lymphoma-2 (Bcl-2, antiapoptotic protein) and lead to the conversion of Bcl-2 from a protector to a killer which triggers the release of cytochrome c and apoptosis [51]. For inflammation, the NR4A receptors are involved in a negative feedback mechanism to maintain the inflammatory balance [105, 106]. In neurological diseases, the NR4A receptors have been involved in relevant neuronal functions and mediate cAMP-response element-binding protein- (CREB-) dependent neuroprotection [107]. In cardiovascular diseases, the NR4A receptors are induced by a wide range of stimuli in vascular cells and have been found to induce atherosclerosis lesions in human subjects and experimental models [108]. In immune response, NR4A1 also takes part in the development and maturation of monocytes [109, 110].

## 5. Conclusions

In summary, orphan nuclear receptor NR4A1 is an immediate-early response gene that is rapidly induced by a variety of stimuli, including multiple stress reaction compounds, growth promoters, cytokines, and miRNA. Increasing evidence implicates NR4A1 activity in the transcription of genes involved in glucose homeostasis, lipid metabolism, and energy balance. Structural studies on the LBDs of NR4A1 revealed that no appropriate ligand-binding cavity is used to bind compound ligands and regulate the physiological and regulatory activities of NR4A. However, several recent studies have identified structurally diverse compounds that bind and activate or inactivate nuclear NR4A1. These compounds show some promise for the treatment of glucose and lipid metabolism diseases. Future studies should clarify the exact role of NR4A1 receptor in metabolism diseases, including obesity, dyslipidemia, and cardiovascular disease, and obtain more novel and effective small molecular compounds based on the structural of NR4A1.
